# Study on Formability and Microstructure Evolution of Usibor^®^2000 Sheet Under Different Forming Temperatures in Deep Drawing Process

**DOI:** 10.3390/ma18102224

**Published:** 2025-05-12

**Authors:** Yuxuan Wang, Jinyu Hou, Peiran Deng, Yongze Shi, Jiacheng Song

**Affiliations:** School of Materials Science and Engineering, Shanghai University of Engineering Science, Shanghai 201620, China

**Keywords:** ultrahigh strength steel, forming temperature, sheet metal formability, deep drawing, thermomechanical processing

## Abstract

Due to the need for weight reduction in the automobile structure, effective and accurate forming is demanded to take advantage of ultrahigh-strength steels. Research on the deep-drawing formability of Usibor^®^2000 has an important impact on the application of lightweight automotive bodies. The microstructure and formability of Usibor^®^2000 sheets at different temperatures were investigated by the Swift test. The positive effects of increasing the temperature on improving the forming limit and forming quality of Usibor^®^2000 were demonstrated by LDR results, thickness, and hardness measurement. The microstructure evolution of Usibor^®^2000 steel plates under warm forming and hot forming conditions was discussed in terms of microstructure characterization and precipitate morphology. The phase composition of the sample deformed at 860 °C is analyzed by two-step etching metallographic analysis and numerical simulation, which provides a reference for the application of Usibor^®^2000 ultrahigh-strength steel in automotive lightweight.

## 1. Introduction

With the increasingly stringent automobile emission regulations around the world and the continuous demand for vehicle impact resistance, higher requirements are put forward for advanced steel in both automotive lightweight and safety [1]. Ultrahigh strength steel (UHSS), an advanced steel with a strength grade higher than 1500 MPa, has outstanding advantages in strength, toughness, wear resistance, and other aspects. Various steel manufacturing enterprises have paid great attention to the development of UHSS in recent decades, such as 22MnB5 developed by SSAB of Sweden [2], Usibor^®^1500 by ArcelorMittal of Luxembourg [3], and B1500H by Baosteel of China [4].

ArcelorMittal’s new generation of UHSS, Usibor^®^2000, has broad application prospects due to its superior resistance and mechanical properties [5]. However, UHSS frequently experiences issues with shape and dimensional stability during the forming process, including cracking and springback, resulting in great difficulties in installation and debugging. Solving the problem of improving the formability and quality of UHSS is a key challenge to promote its broad application. Hot forming technology has become an effective approach to achieve efficient and high-quality processing of UHSS due to its superior formability and low flow stress at high temperatures [6,7,8].

The profitable hot forming process of UHSS has received great enrichment and optimization in response to the large and rapidly growing market demand for steel applications. The rich variety of research results on the hot forming technology of UHSS has been achieved in the heating method [9,10,11], process window [12,13,14], numerical simulation [15,16], and microstructure evolution [17,18]. The majority of research on Usibor^®^2000 processing and application has only focused on the constitutive model [19], soft zone design [20], and thermal processing process window [21], and failed to address the microscopic deformation mechanism of Usibor^®^2000 under the complex action of temperature and force. The plasticity and formability of Usibor^®^2000 in the hot forming process are superior to those in the warm forming process, due to not only the occurrence of deformation and recrystallization, but also the evolution of the second precipitated phase [22]. Recent studies indicate that the matrix of commercial 38MnB5 was effectively reinforced through the uniform dispersion of (Nb, Ti)C nanoparticles as a second phase [23]. Nevertheless, the phase transition law and the second phase precipitation behavior in Usibor^®^2000 steel still call for an explanation in detail. The mechanical properties and formability of UHSS such as Usibor^®^2000 are intricately linked to its microstructural constituents and alloying element distribution. The steel’s high strength is primarily attributed to the presence of hard martensitic phases, which are significantly affected by its chemical composition, including carbon, boron, and microalloying elements such as Nb, Ti, and Mo. These elements influence the austenite stability, delay phase transformations, and promote precipitation strengthening via (Nb,Ti)C or (Ti,Nb)(C,N) carbides. During thermomechanical processing, these factors govern the kinetics and morphology of phase transitions, especially the competition between ferrite, bainite, and martensite formation depending on cooling rates. Understanding these mechanisms is essential for optimizing forming processes at various temperatures. Furthermore, modeling these phase transformations in real deep-drawing conditions provides valuable predictive insights for practical manufacturing, allowing for virtual process tuning and reducing the cost of trial-and-error experimentation. However, comprehensive studies linking microstructural evolution, mechanical response, and numerical simulations of thermal-mechanical behavior under deep drawing conditions remain scarce, particularly for Usibor^®^2000.

Despite recent advances in processing technology and phase transformation modeling of Usibor^®^2000, the fundamental question of how microscopic deformation mechanisms and precipitate evolution govern formability across different thermal regimes remains unresolved. Therefore, this study hypothesized that the formability and final mechanical properties of Usibor^®^2000 in the deep drawing process could be significantly influenced by temperature-induced microstructural evolution, particularly through the behavior of second-phase precipitates. The main objective was to elucidate the thermomechanical deformation mechanisms of Usibor^®^2000 under different forming temperatures, integrating microstructural characterization, phase composition analysis, and numerical simulation to bridge the gap between macro-scale forming outcomes and underlying microstructural processes.

## 2. Numerical Simulation

**Figure 1:**
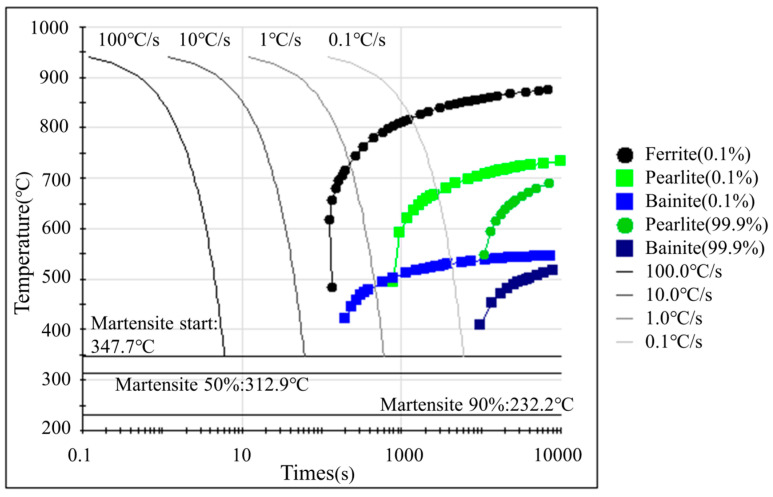
CCT curve of Usibor^®^2000.

The phase transition behavior in sheet metal during the hot forming process was calculated theoretically to study the deformation mechanism of Usibor^®^2000 and analyze the phase composition evolution. The CCT curves for Usibor^®^2000 steel, provided by the JMatPro software v7.61 in Figure 1, show that varying cooling rates lead to different phase compositions. As the cooling rate increases, the martensitic content in the transformed microstructure also rises. The critical cooling rate required to achieve a fully martensitic phase falls between 1 °C/s and 10 °C/s. However, coupled with the in-die heating system, the cooling method adopted in this study basically relies on mold pressure cooling quenching, which is relatively slow, and fails to achieve the full martensitic transformation of Usibor^®^2000 in the hot forming process.

**Figure 2:**
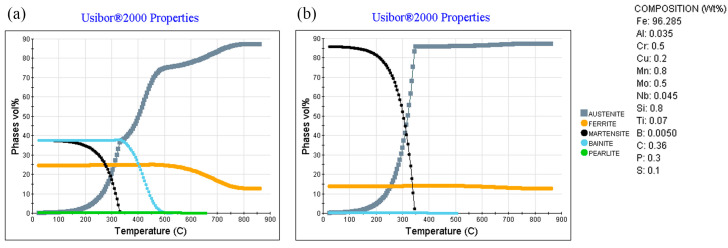
Usibor^®^2000 quenching phase transition at different cooling rates at the austenitizing temperature of 860 °C (**a**) 1.0 °C/s (**b**) 10 °C/s.

In this study, the cooling phase transformation of Usibor^®^2000 steel during the hot stamping process at an austenitizing temperature of 860 °C is illustrated in Figure 2 for two different cooling rates: 1 °C/s and 10 °C/s. Because the initial sheet temperature did not reach the austenitizing temperature of the material, 86.89% of the initial structure is austenite, with the remaining 12.62% ferrite, according to JMatPro. The ferritic transition begins at 800 °C and slowly increases, eventually stopping at 500 °C. At this point, the bainite transition begins and continues until it stops at 340 °C and then turns to a martensitic transition until almost all of the austenite is transformed. At a cooling rate of 1.0 °C/s, the final microstructure consists of 37.53% martensite, 24.66% ferrite, 37.46% bainite, and 0.35% residual austenite. In contrast, when the cooling rate is increased to 10 °C/s, the rapid temperature drop prevents austenite from transforming into ferrite and bainite. Instead, it reaches the martensitic transformation temperature range, resulting in the formation of martensite. Consequently, at a cooling rate of 10 °C/s, the final microstructure shows a significant shift compared to that at 1.0 °C/s, comprising 85.85% martensite, 13.85% ferrite, 0.17% bainite, and 0.14% residual austenite. The martensite content of Usibor^®^2000 after press hardening should be within the range of these two conditions, and the specific content and proportion need to be determined on the formed samples.

**Figure 3:**
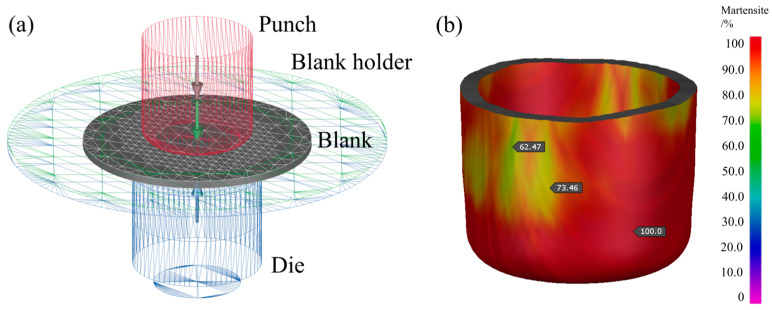
Numerical simulation results of deep drawing Usibor^®^2000 (**a**) Deep drawing mold structure (**b**) Martensitic phase distribution.

The numerical simulation analysis of the Swift cylinder drawing process in the 860 °C hot forming scheme was carried out by Autoform R8 software. The specific geometric model and mesh division of the deep drawing die are shown in Figure 3a. Under ideal conditions, the martensitic structure is formed in most areas of the sheet metal after thermoforming cooling; especially, the rounded corner area is the best quenching effect and the complete martensitic structure can be achieved, as shown in Figure 3b.

To validate the simulation results, the predicted phase fractions were compared with experimental data obtained from a two-step color metallographic analysis. The simulation estimated a martensite content ranging between 37.53% and 85.85% depending on the cooling rate, and the experimentally measured martensite content in the 860 °C formed samples was 65.89%, which falls within the predicted range, demonstrating good agreement between the simulation and the experiment.

Although in situ techniques such as dilatometry or thermal imaging were not employed in this study, the boundary conditions for the numerical simulation were carefully defined based on the actual processing parameters used in the thermoforming tests, including the austenitizing temperature, holding time, and in-die cooling method. Cooling rates of 1 °C/s and 10 °C/s were selected to represent the lower and upper bounds of industrial press hardening conditions. These parameters ensured that the simulation results closely reflect the expected microstructural transformations during forming.

## 3. Materials and Methods

**Figure 4:**
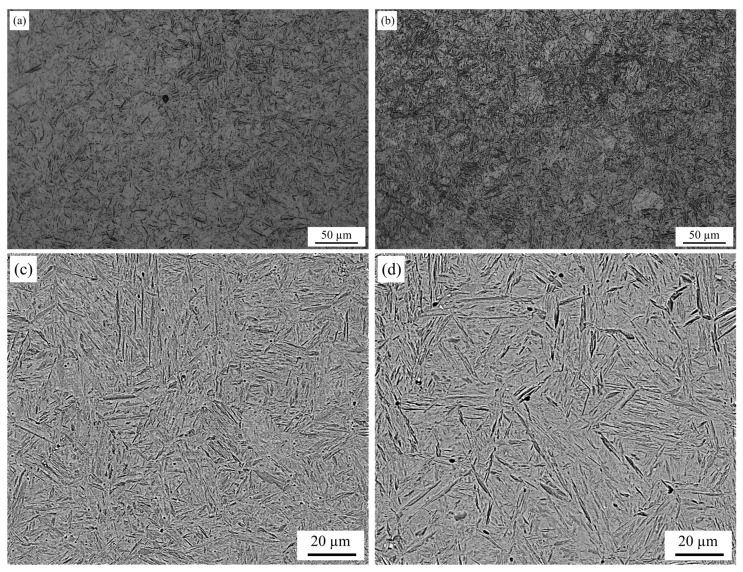
(**a**,**b**) OM and (**c**,**d**) SEM images of martensitic tissue obtained from Usibor^®^2000 quenching after holding at 950 °C (**a**,**c**) for 5 min and (**b**,**d**) for 10 min.

The experimental material used in this study is an as-received Usibor^®^2000 steel sheet (ARCELORMITTAL, Luxembourg) with a thickness of 1.8 mm. Its chemical composition (in weight percentage) is Fe-0.36C-0.19Si-0.8Mn-0.28Cr-0.36Mo-0.04Nb-0.0032B. According to JMatPro software, the calculated austenitizing temperature is 952.18 °C. Therefore, the sheet was heated to 950 °C and maintained for 5 and 10 min before quenching. The resulting martensitic structures are illustrated in Figure 4a,b. It can be observed that the ferrite can be completely transformed into austenite, and then formed into a martensitic structure through a quenching and cooling transformation. By comparison, the uniform microstructure of martensite was obtained after a phase transition when the holding time is 10 min, as austenite has more time to form and grow.

Figure 4c,d shows SEM images of martensitic structures obtained after Usibor^®^2000 was held at 950 °C for 5 min followed by a quenching duration of 10 min. In both images, the martensite slats are arranged parallelly in a bundle and group, as well as the interlaced sheet martensite in different sizes, which constitute the mixed martensitic structure. The coarser-lath martensite groups and longer-sheet martensite are obtained when the material is held at an austenitizing temperature for 10 min, as shown in Figure 4d.

**Figure 5:**
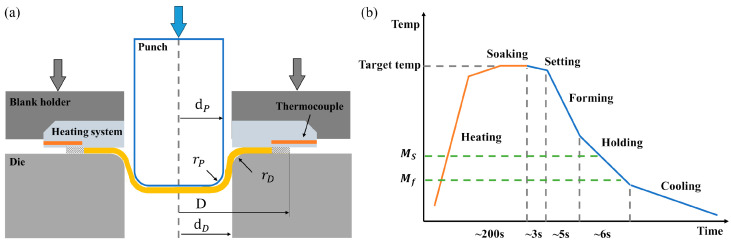
(**a**) Die structure and (**b**) time-temperature changing curve for one shot.

The formability for the Usibor^®^2000 sheet at different temperatures was evaluated using a KOMATSU H1F60 servo press (KOMATSU, Komatsu, Ishikawa, Japan) equipped with an in-die heating system. Figure 5a illustrates the die setup used for the Swift cup tests, while Figure 5b presents an approximate timeline of the thermoforming process across various temperature conditions. According to the GB/T 4156–2020 standard [24], the limiting drawing ratio (LDR) is defined as the ratio between the limiting blank diameter (D) and the punch diameter ($d_{p}=2$6.24 mm). Additional details regarding the testing procedure and parameters are available in previous work [25]. Deep drawing experiments were conducted within a temperature range of 100 °C to 860 °C, using 100 °C intervals. The stamping speed is set at 40 mm/s, and the blank holder force is maintained at 8 MPa.

The thickness profile of the formed cups was evaluated by taking an average of three measurements at each point. A digital Vickers microhardness tester was used, applying a 50 g load for 10 s. Microstructural analysis was conducted using a scanning electron microscope (SEM, Zeiss Gemini Sigma 300, Carl Zeiss AG, Oberkochen, Germany), and the elemental composition of the precipitates was identified using an energy-dispersive spectroscopy (EDS) system.

The microstructure of the drawn sample was characterized by two-step color metallographic etching. This technology was developed by De et al. [26] to identify different metallographic structures and the distribution of each phase by different colors after corrosion and coloring in multiphase steel. Bardelcik and George et al. [27,28] also employed this method to analyze the phase composition and friction distribution in hot-stamping processes.

The color metallurgical etching analysis is carried out in two stages. Initially, a solution of 4% picric acid (4 g dissolved in 100 mL of ethanol) is mixed with 1 mL of hydrochloric acid. This mixture is then used to etch the polished sample for around 20 s, and then immediately clean the sample with a large amount of water and wipe it with alcohol. After that, the sample is etched for roughly 10 s using a 10% sodium metabisulfite solution. Under the metallography microscope, the sample’s martensite will appear yellowish brown, the bainite black, and the ferrite white [29]. The different color proportions can be calculated and analyzed by image processing software ImageJ 1.53k through image region and pixel statistics to distinguish the phases in the metallographic photos. In this paper, martensite is marked red, ferrite is marked blue, and bainite is marked green.

### Lubrication and Tooling Surface Treatment

To minimize interfacial friction during the deep drawing process, a high-temperature solid lubricant, specifically a boron nitride-based coating, was applied to both the punch and die surfaces before each forming test. This lubricant was selected for its effectiveness in high-temperature applications and its ability to reduce direct metal-to-tool contact. Additionally, the forming tools were polished to a surface roughness (Ra) of less than 0.2 μm to further reduce frictional resistance and ensure consistent material flow during deformation. These lubrication and surface treatment conditions were maintained consistently throughout all tests to ensure uniformity in the experimental setup.

## 4. Results and Discussions

### 4.1. Formability Analysis

**Figure 6:**
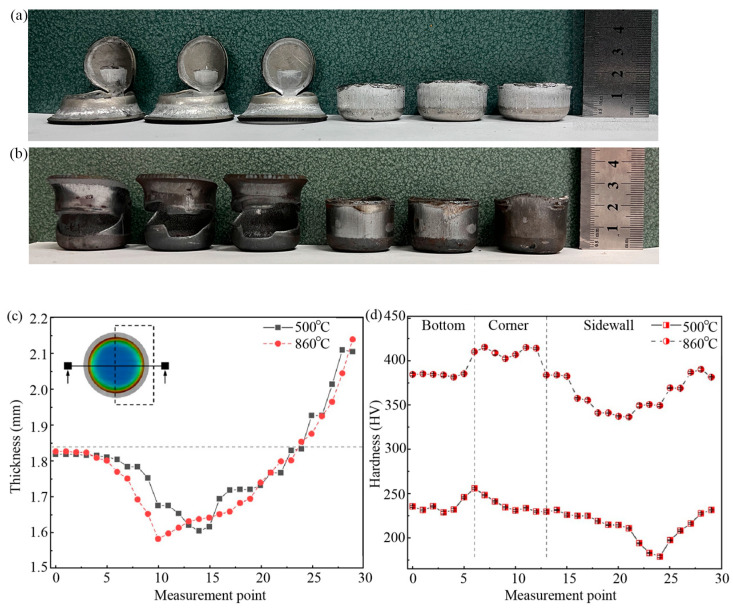
Cup height of drawn samples at (**a**) 500°C and (**b**) 860°C. (**c**) Thickness distribution of molded cylinders at different temperatures (**d**); hardness distribution of molded cylinders at different temperatures. The interval of warm and hot forming cylindrical parts was 0.75 mm and 0.85 mm, respectively.

To analyze the influence of temperature on the drawing process of the Usibor^®^2000 sheet, Figure 6a,b show a comparison of the cup height formed at 500 °C and 860 °C, respectively. The height of the cylindrical part formed at 860 °C increases by 53% compared to that at 500 °C. The LDR value at 860 ℃ was 2.05, which was 16.5% higher than that at 500 ℃ (1.76).

The thickness distribution of formed samples under drawing temperatures was assessed, as illustrated in Figure 6c. The maximum thinning area is located at the tangent point between the cylinder wall and the rounded corner of the drawn cup, which is in good agreement with the splitting position in the broken parts. The similar curves of thickness distribution indicate that the drawing performance of sheet metal is improved under the condition of hot forming. However, it can be seen from Figure 6d that the hardness of the hot drawing formed part is almost twice that of the warm drawing due to the martensitic phase transition, which means that the hot forming part obtains more ideal strength and forming quality.

**Figure 7:**
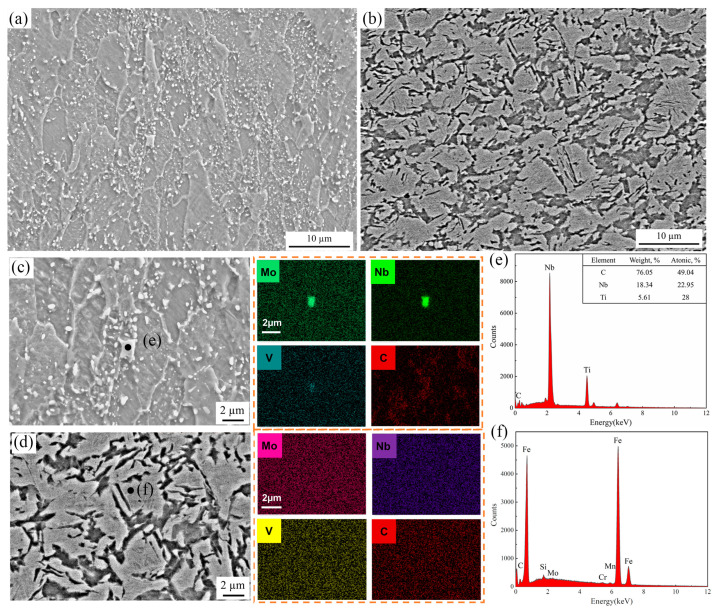
SEM and EDS images of samples formed at (**a**,**c**) 500 °C and (**b**,**d**) 860 °C, and the top right corner is images for elemental mapping. (**e**) Morphology type of the precipitates in (**c**). (**f**) Compositional profile of the matrix in (**d**).

The microstructure and compositional distribution of the specimens were further investigated, as shown in Figure 7, on which elemental mapping analysis of Nb, Mo, V, and C was induced, as depicted in Figure 7c,d. The large-size segregation component of Nb, Mo, and a little V, with an average diameter of 1.1 µm, was identified as Nb-rich (Nb, Ti) C carbide that is not solidly dissolved into the matrix during warm drawing at 500 °C, as shown in Figure 7c. The (Ti, Nb) (C, N) carbonitride remaining at 500°C can be considered as the crack source of the forming sheet [30]. As the temperature increases, most of the second phase has been dissolved into the matrix and has no time to precipitate in the form of a large-scale solid solution because of the high rapid cooling. In addition, the small-sized precipitates can be used as the martensitic nucleus substrate to promote the solid solution strengthening of sheet metal during deep drawing at 860 °C [31]. To further support these findings, a quantitative analysis of the precipitates was performed on representative SEM images using ImageJ. For the sample formed at 500 °C, the average diameter of (Nb,Ti)C precipitates was approximately 1.12 ± 0.28 μm, with an estimated number density of ~2.5 × 10^9^ particles/cm^2^. In contrast, precipitates in the 860 °C sample were more dispersed and finer, with an average diameter of 0.38 ± 0.11 μm and a number density of ~6.8 × 10^9^ particles/cm^2^. These quantitative differences further illustrate the enhanced dissolution and redistribution of alloying elements at higher temperatures, promoting a more favorable martensitic transformation and strengthening behavior.

### 4.2. Phase Composition Analysis

**Figure 8:**
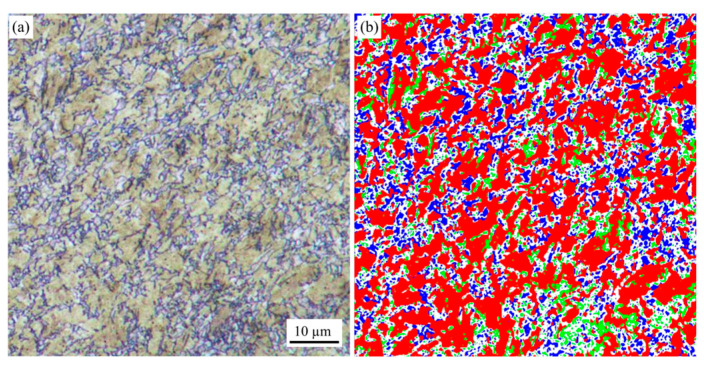
Phase distribution and proportion of the side wall tissue of the specimen drawn at 860 °C (**a**) Color metallography obtained by two-step dyeing (**b**) phase area statistics and phase fractions.

A two-step color metallographic etching technique was employed to analyze the phase distribution and composition within the sidewall structure of the drawn sample formed at 860 °C, as illustrated in Figure 8. The sidewall of the drawn sample formed at 860 °C exhibits a multiphase microstructure due to quenching during the forming process. Color etching reveals the coexistence of martensite, bainite, and ferrite. Martensite is arranged in an irregular, slat-like pattern, while bainite is characterized by a ferrite matrix with dispersed cementite particles [32].

The distribution of martensite, bainite, and ferrite in the sidewall microstructure of the sample drawn at 860°C was quantified through image analysis of the color metallographic image. Based on the processed data shown in Figure 8b, the area fractions for martensite, ferrite, and bainite are 65.89%, 21.25%, and 12.86%, respectively. Some ferrite and bainite phases formed before the martensitic phase transformation, resulting in a mixed microstructure in the sample. This structure, achieved at the cooling rate used in this test, provides an optimal balance of strength, toughness, and enhanced application potential. The result is basically consistent with the predicted martensite content range obtained by numerical simulation calculation.

The experimental results obtained from two-step color metallographic etching showed good agreement with the phase fractions predicted by simulation, particularly in the proportions of martensite, bainite, and ferrite. This consistency indirectly validates the phase transformation behavior modeled in the simulation and supports the use of CCT-based simulation as a reliable tool for predicting microstructure evolution under practical forming conditions.

To further interpret the observed increase in hardness at 860 °C, the hardness contribution of each phase was estimated using a rule-of-mixtures approach. Based on literature values, the Vickers hardness of martensite, bainite, and ferrite in boron-added ultrahigh strength steel is approximately 550 HV, 350 HV, and 200 HV, respectively [33]. Using the measured phase area fractions (65.89% martensite, 12.86% bainite, and 21.25% ferrite), the estimated average hardness is:

H_avg_ = 0.6589 × 550 + 0.1286 × 350 + 0.2125 × 200 = 451.3 HV (1)

which agrees well with the experimentally measured hardness (~460 HV). This result quantitatively supports the assertion that martensite formation dominates the observed hardening behavior at higher forming temperatures.

## 5. Conclusions

This study demonstrates that Usibor^®^2000 exhibited significantly improved formability and mechanical properties under hot stamping conditions. Specifically, deep drawing at 860 °C resulted in a limiting drawing ratio (LDR) of 2.05–16.5% higher than that at 500 °C— and a 53% increase in cup height. The enhanced performance could be attributed to the formation of a predominantly martensitic microstructure (65.89%), complemented by bainite and ferrite, as confirmed by two-step color metallography and consistent with numerical predictions. Compared to conventional press hardening steels, such as 22MnB5, Usibor^®^2000 demonstrated superior hardenability and strength-formability synergy due to its alloying with Nb, Mo, and Cr, promoting refined and stable austenite formation during heating and efficient martensitic transformation during quenching. Additionally, its capacity to achieve uniform phase distribution and solid solution strengthening under optimized forming conditions makes it a strong candidate for automotive structural components requiring high crashworthiness and weight reduction. These findings highlight the practical potential of Usibor^®^2000 in advanced hot forming applications and contribute to the ongoing efforts in lightweight, high-strength automotive design. It is acknowledged that the inclusion of temperature-dependent uniaxial tensile tests could further clarify the relationship between microstructure evolution and mechanical properties. Future research should concentrate on integrating such data to enhance the understanding of the thermal-mechanical response of Usibor^®^2000 during forming. Future research integrating temperature-dependent tensile testing and simulation will further elucidate the microstructure–property–process relationships in this material system.

## Data Availability

The original contributions presented in this study are included in the article. Further inquiries can be directed to the corresponding author.
